# Early Approach Towards Atypical Guillain-Barré Syndrome: A Physiotherapy Perspective in a Case Report

**DOI:** 10.7759/cureus.31235

**Published:** 2022-11-08

**Authors:** Jaee P Kapre, Pallavi Harjpal, Snehal S Samal

**Affiliations:** 1 Department of Physiotherapy, Ravi Nair Physiotherapy College, Datta Meghe Institute of Medical Sciences, Wardha, IND; 2 Department of Kinesiology, Ravi Nair Physiotherapy College, Datta Meghe Institute of Medical Sciences, Wardha, IND

**Keywords:** neurological rehabilitation, early physiotherapy, rehabilitation, atypical gbs, guillain-barré syndrome

## Abstract

Guillain-Barré syndrome (GBS) is acute, ascending, immune-mediated, monophasic polyneuropathy, which manifests itself as a lower motor neuron lesion, which occurs mostly after a prior infection. It is autoimmune in origin and has an impact on the peripheral nervous system. GBS is usually not linked to an autoimmune or other systemic condition and is most frequently a post-infectious disorder that affects healthy patients. The symptoms of GBS, an acute immune-mediated polyradiculoneuropathy, include symmetrical limb weakness that worsens quickly and hypo- or areflexia. There may also be sensory complaints, involvement of cranial and autonomic nerve fibres, and frequent pain that will appear before weakening. Weakness, sensory loss, weariness, and discomfort are the most typical remaining deficiencies in an atypical variant of GBS. This case also describes the variant of atypical GBS. A 10-year-old girl was referred to the hospital with complaints of difficulty in swallowing, drooling of saliva, weakness of left upper and bilateral lower limbs, and fever for 10 days. There was no past history of travelling or infection. At the time of admission, the patient was on oxygen support for breathing and she was transferred to ICU immediately. Investigations were done such as a nerve conduction velocity test and complete blood count. Neuro-physiotherapy of the patient was started after 35 days of hospitalisation. With proper rehabilitation, the patient was able to gain strength and the ability to swallow food. The patient was able to resume her academic career.

## Introduction

Guillain-Barré syndrome (GBS) is a polyneuropathy with an acute immunological origin. In the most advanced stages of the illness, about two-thirds of patients having GBS are unable to walk. The two causes of mortality are autonomic dysfunction and inadequate respiratory function. The cranial nerve variety affects few patients, which is most typically found in Miller-Fisher syndrome (MFS), and even fewer patients have the pure motor form [1]. GBS often starts suddenly with paresthesias that develop distally and very symmetrically. The four main subgroups of this illness are MFS, acute inflammatory demyelinating polyradiculoneuropathy (AIDP), acute motor axonal neuropathy (AMAN), and acute motor and sensory axonal neuropathy (AMSAN). Patients with a confirmed diagnosis of GBS will get IV immunoglobulin (IVIG) or plasmapheresis treatment. There are 1.2-2.3% cases of GBS per 100,000 people per year, according to reports. For every ten years of age increase, the incidence rises by 20%. Four studies conducted in Western nations indicate a winter high, while those conducted in northern China, India, Bangladesh, and Latin America indicate a summer peak [2,3]. Most of the time, a post-infectious illness is GBS, which typically affects healthy individuals. The primary symptom of GBS is quickly developing bilateral and symmetrical limb weakness, whether or not respiratory muscles or muscles with cranial nerve innervation are involved. Patients suspected of having GBS always have a lumbar puncture performed. A normal CSF white cell count and increased protein are frequently seen during a CSF test [4]. Dysphagia, bilateral vocal cord paralysis, optic neuritis, and hearing loss can all be brought on by cranial nerve involvement. In the first 12 weeks after an acute episode, 30% of them may have pneumonia and respiratory failure. In the preliminary stages of the condition, aggressive respiratory treatment with pulmonary hygiene is required, as well as acute inpatient rehabilitation. Due to aspiration, patients with cranial nerve damage are more vulnerable to pulmonary infections. Perhaps, for this reason, the severity of GBS and ventilatory dependency have been strongly correlated with cranial nerve damage [5]. Weakness, sensory loss, weariness, and discomfort are the most typical remaining deficiencies [6]. Previous studies have demonstrated the effectiveness of cardiovascular exercise, neurorehabilitation such as active-assisted training, and then strengthening [7].

## Case presentation

Patient information

As narrated by the parents, a 10-year-old girl was referred to our hospital from a private hospital with oxygen support attached to her. Her presenting complaint was difficulty in breathing with sputum and cough production, difficulty in swallowing with drooping of saliva, neck pain, and weakness in the left upper and bilateral lower limbs with dizziness. The Hughes severity scale score was five. The patient was apparently fine one month back when she went to school and came back home complaining of neck pain, difficulty in swallowing food, and a cough. She was then taken to a local hospital where consultation and medications were given by the doctor; she was hospitalised for four days for the same. For further management, she was taken to a private hospital in Amravati, India, where she had a spike in fever for one day and was admitted to the ICU for five days under observation. But due to limited facilities, she was referred to Acharya Vinoba Bhave Rural Hospital (AVBRH), Wardha, India, for further treatment, where she had a fever for five more days with drooling of saliva and was under observation in the ICU. Then, the patient was referred to physiotherapy after 15 days of her initial symptoms when the patient was hemodynamically stable.

Clinical findings

The patient had a mesomorphic build and was aware and well-oriented. She exhibited both bilateral upper and lower extremity sensations that were intact. With a grade of 3/5 on the Medical Research Council muscle scale in the shoulder, 3+/5 elbow, and 3+/5 wrist, a grade of 2/5 in the hips and 3/5 in the knee muscles, and a score of 3+/5 over her ankle muscles, muscular strength was reduced in the left upper and bilateral lower limbs suggesting weakness. Lower limbs were more involved than upper limbs. Lower limb deep tendon reflexes were reduced, whereas bilateral biceps, triceps, and supinators had retained reflexes. Plantar reflexes were missing on both the left and right side, bowel and bladder function were unaffected, and abdominal reflexes were normal. Breathing was normal with secretions, but she had difficulty taking deep breaths and was using her accessory muscle (sternocleidomastoid).

Clinical diagnosis

The patient had undergone a nerve conduction velocity (NCV) study, which showed sensory-motor polyneuropathy. Compound muscle action potential (CMAP) amplitude could not be elicited in the bilateral median, ulnar, tibial, and peroneal nerves. Sensory nerve action potential (SNAP) amplitude could not elicit bilateral median, ulnar, and sural nerves. A complete blood count was done and a blood swap for culture was also taken. The timeline of events in the ICU, Ward, and Neuro Rehab, is shown in Table 1.

**Table 1:** Timeline of events NBM: nil by mouth; NS: normal saline; NP: nasal prong; SDS: sodium dodecyl sulfate; CPAP: continuous positive airway pressure; PEEP: positive end-expiratory pressure; RT: Ryles tube; ADL: activities of daily living; IADL: instrumental activities of daily living

Sr.no	Date of Events	Consultation	Findings	Suggestions
1	On admission (Patient was shifted to the hospital with a 5L Of O_2 _cylinder)	Casualty	Difficulty in swallowing, drooling of excessive saliva, weakness in the bilateral upper and lower limbs. Difficulty in breathing.	NBM IV fluid of 4ml KCl for every 12 hourly (100%); Injection meropenem 500mg in 50 ml NS over 2 hours IV for every 8hrs (120mg/kg/dose); Injection vancomycin 375mg in 50 ml NS over 2 hrs IV for every 6hrs; Injection PAN 25mg IV for every 2 hrs (1mg/kg/day); Injection Emset 4mg IV (0.15g/kg/dose)
2.	19/08/2022	Paediatric ICU	Fever Spike to 100.4 ^0^F, maintaining saturations	O_2_ by NP at 2l/min; Injection DNS 400ml; Injection KCL 4ml in E/D IV every 8hr (100%); Injection meropenem 500mg in 50ml NS over 2hrs IV every 8hr (20mg/kg/dose); Injection vancomycin 400mg in 50ml NS over 2hrs IV every 6hrs (15ml/kg/dose); Injection Pantop 25mg IV every 24hrs (1mg/kg/dose); Injection Emset 4mg IV every 8hr (0.15mg/kg/dose)
3.	20/08/2022	Paediatric ICU	For maintaining saturation and difficulty in breathing	O_2_ by CPAP: PEEP: 6mm Hg, flow rate: 6L/min; Injection Neomol 375mg/38ml IV SDS (15mg/kg/dose) Call for ophthalmology no abnormality was present.
4.	22/08/2022	Paediatric ICU	Respiratory distress, with fluctuations in saturation	RT feeds 50ml/2hrs (600ml); 2L/min; Air entry was decreased on the right side and crepitation’s present on the left side.
5.	23/08/2022	Paediatric Ward	Atypical Guillain-Barré syndrome (GBS) with involvement of cranial nerve	The physiotherapy session of the patient started on 23/08/2022 and continued till the patient got discharged.
6.	132/09/2022	Neuro Physiotherapy	Weakness of bilateral upper and lower limb; swallowing difficulty	Strengthening exercises, breathing exercises, and fine motor exercises.
7	21/09/2022	Neuro Rehab	Difficulty in performing ADLs and IADLs; swallowing difficulty	Strengthening exercises to bilateral upper and lower limbs, gait training, fine motor activities and oro-pharyngeal stimulation.

Physiotherapy functional assessment

After 35 days with GBS-related symptoms, the secretions were present on the left side and air entry was reduced on the right side for which chest physiotherapy was given (breathing exercises, manual chest percussions and vibrations, passive end-expiratory pressure, pursed lip breathing and position) was given to the patient. To prevent bed sore and maintain the mobility of the patient, bed positioning with pillows every two hours was taught to the patient’s caregiver. The following treatment protocol was given once a day for 10 repetitions of one set with the proper amount of rest. Also, to avoid fatigue in the patient proper number of intervals was provided. The level of functional independence measure of this patient was two and the Hughes severity scale score was five. The patient was moderately dependent on caregivers for her ADLs such as supine to side lying, sit to stand, toileting bathing. Intervention is as follows (Table 2). 

**Table 2:** Physiotherapy interventional protocol

Sr. No	Problems identified	Cause of problem	Interventions
1.	Reduced air entry in the right side of the lung	Weakness of the primary ventilatory muscles (intercostal muscles and diaphragm)	Pursed lip breathing, incentive spirometry and thoracic expansion exercises with manual resistance.
2.	Crepitations were heard over the left side of the lung	Due to the congestion in the left lobe of the lung.	Manual chest percussions and vibrations, passive end-expiratory pressure, pursed lip breathing along with position.
3.	Decreased Range of motion	Due to prolonged immobilization.	Active Range of motion exercises to the bilateral upper and lower limbs.
4.	Swallowing difficulty	Involvement of cranial nerve (Glossopharyngeal)	Oro-motor stimulation, blowing of paper, air gulping exercises, facial muscle exercises, positioning food in an optimal position to stimulate gulping pharyngeal electrical stimulation 200 μs pulses at 5 Hz, the frequency was less than 1/min) [8]
5.	Difficulty in speaking	Cranial nerve involvement (Hypoglossal)	Speech training, oro-motor facilitation.
6.	Reduce muscle strength	Decreased nerve conduction, prolonged bed rest and disuse atrophy.	To give electrical muscle stimulation to the weakened muscles. Bilateral upper limb strengthening training (1/2L water bottle and progress to 1L) Bilateral lower limb strengthening training (1/2kg weight cuff and progress to 1kg weight cuff) (Figure 1A-1D)
7.	Decreased bed mobility	Weakness and decrease in the endurance of muscle.	Rolling facilitation and transition training (supine to sit, pelvic bridging, unilateral bridging), Pelvis PNF (Figure 2A, 2B)
8.	Reduced out-of-bed transition	Weakness of pelvis girdle muscles and reduced strength	Transition training, supine to sit, sit to stand
9.	Impaired standing dynamic balance	Prolonged bed rest	Star-excursion exercises, obstacles walking, Proprioceptive training.
10.	Impaired fine motor training	Distal muscle weakness, cognition impairment	Pegboard activities, handwriting activities, rubber band exercises.
11.	Balance training	Prolonged hospital stays	Dual-task training, dynamic standing balance with perturbations, dynamic standing balance with reach-outs, tandem walking, asking the patient to pick up the objects from the floor and trunk rotation exercises. (Figure 3A, 3B)

**Figure 1:**
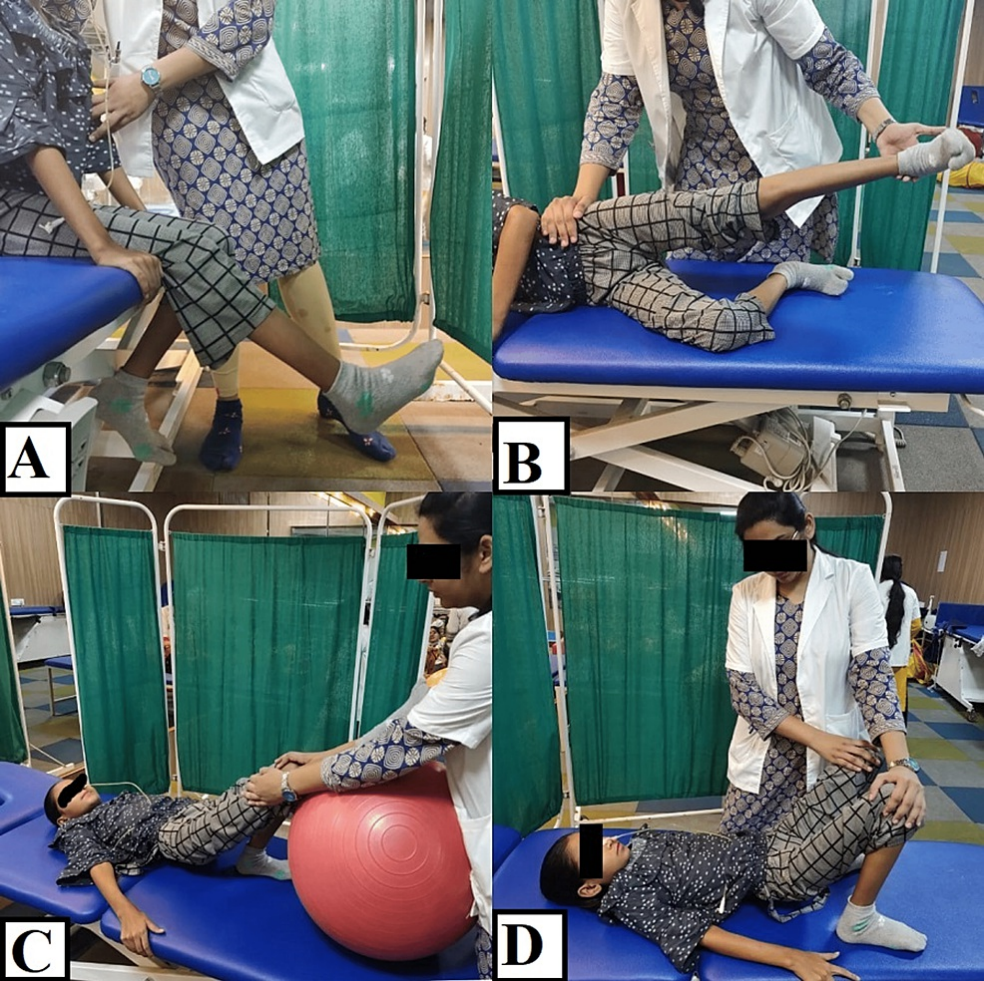
Patient performing exercises A: Dynamic quadriceps strengthening; B: Abductor strengthening exercises; C: Unilateral bridging with help of a swiss ball; D: Pelvic bridging exercise

**Figure 2:**
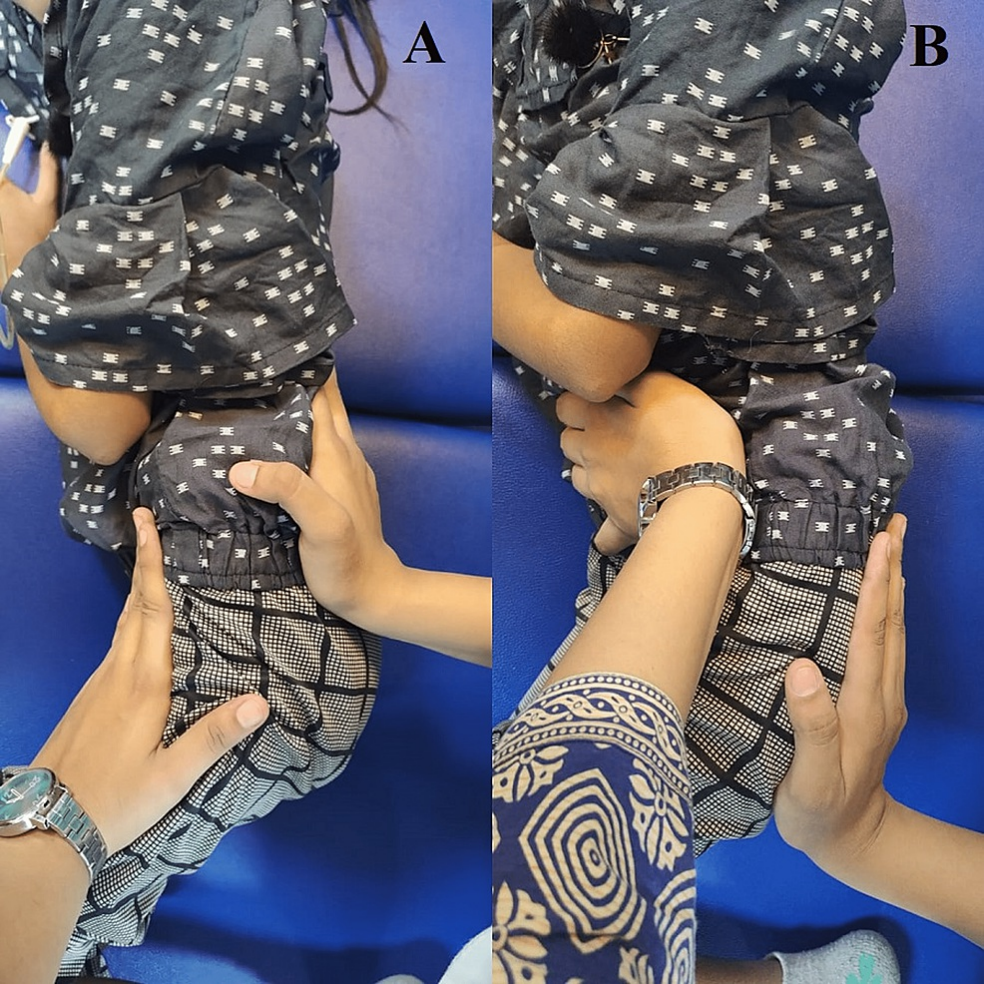
Showing Pelvic proprioceptive neuromuscular facilitation using slow reversal A: Anterior elevation; B: Posterior depression

**Figure 3:**
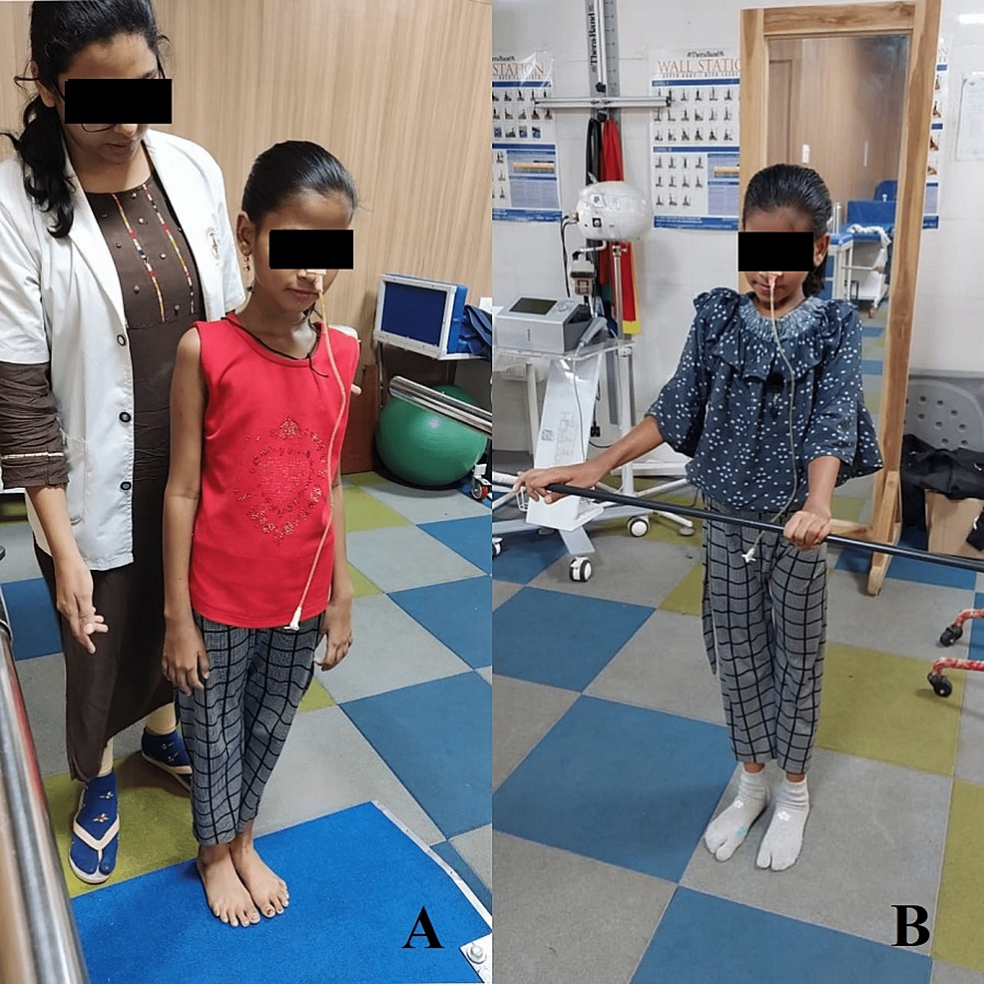
Patient training A: Balancing training; B: Standing with the narrow base of support

Figure 1 shows the patient performing the various exercises. Figure 2 shows pelvic proprioceptive neuromuscular facilitation using slow reversal. Figure 3 shows patient training.

Follow-up and outcome measures

**Figure 4:**
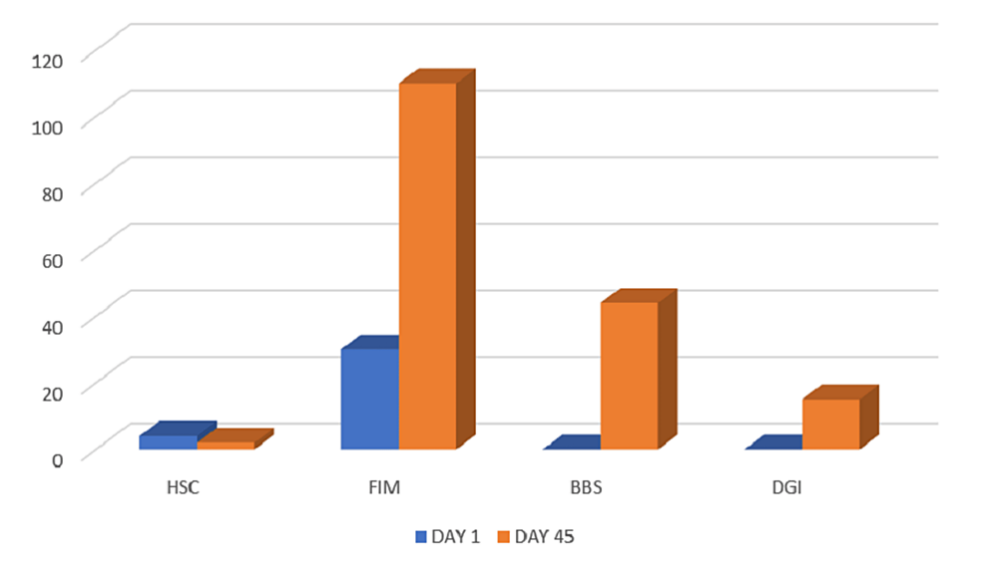
Outcome measures on day one and day 45 HSC: Hughes Severity Scale, FIM: Functional Independence Measure, BBS: Berg Balance Scale, DGI: Dynamic Gait Index

There was an improvement in the manual muscle testing (MMT) score (Table 3) and the outcome measures, i.e., the Hughes severity scale, functional independence measure, and Dynamic Gait Index (Figure 4).

**Table 3:** Manual muscle testing (MMT) score pre- and post-rehabilitation

MMT	Pre-Rehabilitation		Post-Rehabilitation	
	Right	Left	Right	Left
Shoulder	3+/5	3/5	4/5	4/5
Elbow	3/5	3+/5	5/5	5/5
Wrist	3+/5	3+/5	5/5	4/5
Hip	2/5	2/5	4/5	4/5
Knee	3/5	3/5	5/5	4/5
Ankle	3+/5	3+/5	5/5	5/5

## Discussion

Peripheral nerve demyelination is a hallmark of the immune-mediated illness in GBS. It displays characteristic sensory abnormalities, diminished or deep tendon reflexes are missing, and ascending symmetrical muscular weakening is there [3]. The severity of fatigue typically appears to be correlated with the severity of the disease, with the probable exception of exhaustion that occurs in a monophasic syndrome like GBS [9]. It is the primary disease-related cause, with 1-2 instances per 100,000 people. a significant drawback since this sort of neurological condition involves bulbar involvement and the inability to lift the head [10]. Out of 33 patients with normal GBS, five children aged 13 months to 15 years had atypical GBS and GBS variant presentations [11]. Furthermore, oropharyngeal dysphagia in GBS patients was probably more common due to cranial nerve damage, and this cohort may also have subclinical dysphagia [12]. Berg balance scale is one of the criteria for discharging patients from rehabilitation who are diagnosed with GBS [13].

In this case report, we have described a patient who has GBS with cranial nerve involvement and trouble swallowing. The patients experience symptoms that are characteristic of atypical GBS, including dysautonomia, sensory ataxia, areflexia, paraesthesia of the extremities and oropharynx, numbness, discomfort, and weakness [14].

For patients with peripheral neuropathies who underwent physiotherapeutic interventions, both land-based therapy and water-based therapy were helpful in enhancing gait and balance outcomes. The length of the walks, the number of repetitions, and the amount of external support, all steadily increased during both land-based and water physical therapy sessions as long as the patient tolerated it. As described, follow-ups are very essential while implementing any progression in the protocol, and to prevent the recurrence of the symptoms in the patients, they should be keenly observed. Patient follow-ups should be strictly monitored in the present case study [15].

The treatment of GBS is a long-term procedure since the distal muscles of the patients gradually weaken. Management also becomes challenging when cranial nerve and chest issues are involved. Early mobility of the patient, in this case, was not feasible because of a chest issue, and the patient stayed on ventilatory support for a while. However, physiotherapy began as soon as the patient entered the ICU. Chest percussion, chest vibration, pursed lip breathing, and end-expiratory pressure were used to treat chest congestion. The patient is given protocols for strengthening the distal muscles of both their bilateral upper and lower limbs, active range of motion (ROM), bed mobility training, pelvic proprioceptive neuromuscular facilitation, dynamic quads, gait training, oro-motor facilitation, and balance training [5].

## Conclusions

Our study concludes that neuro physiotherapy has proven to be a positive rehabilitation approach in the improvement of functional independence and reducing hospital stay for the patients. Rehabilitation of such patients requires time but proper protocol and regular rehabilitation for the patients makes them able to go to their normal daily life. Further studies will plan a physiotherapy protocol for the management of atypical GBS.
